# Direct Growth of Single Crystalline GaN Nanowires on Indium Tin Oxide-Coated Silica

**DOI:** 10.1186/s11671-019-2870-9

**Published:** 2019-02-05

**Authors:** Aditya Prabaswara, Jung-Wook Min, Ram Chandra Subedi, Malleswararao Tangi, Jorge A. Holguin-Lerma, Chao Zhao, Davide Priante, Tien Khee Ng, Boon S. Ooi

**Affiliations:** Photonics Laboratory, King Abdullah University of Science and Technology (KAUST), Thuwal, 23955-6900 Saudi Arabia

**Keywords:** Nanowires, Gallium nitride, Silica, Indium tin oxide

## Abstract

In this work, we demonstrated the direct growth of GaN nanowires on indium tin oxide (ITO)-coated fused silica substrate. The nanowires were grown catalyst-free using plasma-assisted molecular beam epitaxy (PA-MBE). The effect of growth condition on the morphology and quality of the nanowires is systematically investigated. Structural characterization indicates that the nanowires grow in the (0001) direction directly on top of the ITO layer perpendicular to the substrate plane. Optical characterization of the nanowires shows that yellow luminescence is absent from the nanowire’s photoluminescence response, attributed to the low number of defects. Conductive atomic force microscopy (C-AFM) measurement on n-doped GaN nanowires shows good conductivity for individual nanowires, which confirms the potential of using this platform for novel device applications. By using a relatively low-temperature growth process, we were able to successfully grow high-quality single-crystal GaN material without the degradation of the underlying ITO layer.

## Introduction

Commercially available III-nitride-based devices are mostly reliant on sapphire as the growth substrate, as they can accommodate the growth of GaN with acceptable material quality. However, the challenge in producing large-diameter sapphire substrate while maintaining an acceptable surface quality of the substrate remains an obstacle in scaling up production [1, 2]. A viable alternative to sapphire as a III-nitride growth substrate would be by using silica-based substrate, as they are economically less expensive and widely used in industry and consumer applications. However, as silica-based substrates are inherently non-conducting, non-transparent conducting layer must be used to enable electrical conductivity [3, 4]. Hence, a method to provide simultaneous conductivity and transparency on top of silica substrate becomes very important. We have previously employed thin Ti interlayer as the nanowire nucleation site to provide simultaneous transparency and conductivity [5]. However, as thin layer of Ti is required, the electrical conductivity of the sample becomes limited.

Another possible method for a transparent and conducting substrate is by employing indium tin oxide (ITO) as a GaN nucleation site, as it is transparent and electrically conductive and can be deposited over a large surface area. The ITO technology is already mature, and it has been widely used in various industries for transparent electrodes. The current conventional technique used to manufacture GaN, however, is not compatible with ITO. The high temperature needed to break down the precursors employed in metal organic chemical vapor deposition (MOCVD) growth leads to the degradation of the ITO layer. Thus, a low-temperature GaN growth method capable of producing high-quality material is required. Previous attempts to grow GaN on ITO at low temperature using sputtering and plasma-enhanced chemical vapor deposition (PECVD) have been performed [6–12]. However, low-temperature growth methods typically lead to polycrystalline material and large number of defects.

In this work, we attempt to circumvent this issue through the direct growth of crystalline GaN nanowires on ITO-coated fused silica using plasma-assisted molecular beam epitaxy (PA-MBE). In PA-MBE, active nitrogen species is supplied to the system by breaking the bond between pure N_2_ gas using RF power. Thus, the growth temperature can be significantly lower compared to other GaN epitaxial growth methods, preventing the degradation of the ITO layer. By utilizing GaN nanowires, it is possible to grow high-quality GaN on top of the polycrystalline ITO layer. Because of the strain relaxation and threading dislocation filtering attributed to the high surface to volume ratio of the nanowires [13, 14], the GaN nanowires typically exhibit single crystallinity and no threading dislocation despite the lack of lattice matching between the nanowires and the underlying nanowire nucleation layer [15].

We investigated the morphology of the nanowires and their relation to the underlying ITO layer, the optical characteristics of the nanowires, and the feasibility of using this platform for device applications. Structural characterizations using electron microscopy reveal that the nanowires grow directly on the ITO layer perpendicular to the substrate plane in the c-plane (0001) direction. Photoluminescence measurement gives a good internal quantum efficiency (IQE) value, while yellow luminescence associated with defect is absent from the emission spectrum. Finally, conductive atomic force microscopy (C-AFM) on n-doped GaN nanowires confirms that the nanowires are conductive, highlighting the possibility of fabricating novel devices using the GaN nanowires on ITO platform. From our work, we opened up the potential of growing III-nitride nanowires on top of ITO for device applications where substrate transparency and conductivity is required.

## Methods

### ITO Thin Film Deposition

The ITO thin film used in this experiment was deposited using the RF magnetron sputtering method. The deposition was done at ambient temperature with argon plasma at 60 W RF power, 2.5 mTorr chamber pressure, and 25 standard cubic centimeter per minute (sccm) gas flow rate. Before deposition, the samples are cleaned with standard solvent cleaning using acetone and isopropyl alcohol. Approximately 100-nm-thick ITO thin film was deposited directly on bare silica.

### III-Nitride Nanowire Growth

The GaN nanowire samples are grown using a Veeco Gen 930 plasma-assisted molecular beam epitaxy (PA-MBE) reactor. Before MBE growth, the ITO-coated silica substrate was annealed inside a rapid thermal annealing (RTA) furnace at 650 °C under Ar ambient for 5 min in order to improve the crystallinity of the ITO layer. Before loading into the chamber, the samples are cleaned using a standard solvent cleaning method. The samples undergo subsequent thermal cleaning at 200 °C and 650 °C inside the MBE load lock and preparation chamber in order to remove moistures and other contaminants, respectively.

During nanowire growth, we used a Ga beam equivalent pressure (BEP) value of 1×10^−7^ Torr according to the BFM ion gauge reading. All substrate temperatures are measured using the thermocouple. In order to promote nanowire growth, an initial seeding layer was deposited at 500 °C. After the initial seeding layer deposition, the substrate temperature was raised to 700 °C for the nanowire growth.

### Structural, Optical, and Electrical Characterization

The surface morphology of the ITO layer was investigated using Agilent 5500 SPM atomic force microscopy (AFM) system. The electrical characteristic of the sample was measured by using conductive AFM (C-AFM) in contact mode. To improve electrical contact between the nanowires and C-AFM tip, a Ni/Au layer with 5/5 nm thickness was deposited on top of the nanowires using e-beam evaporation, followed by rapid thermal annealing at 600 °C in atmospheric ambient. The C-AFM measurement was done by using a Pt/Ir-coated AFM tip and applying bias to the ITO layer of the sample. As in our C-AFM configuration bias is applied on the substrate, positive current flow indicates current flowing from the sample to the AFM tip.

The structural quality of the GaN nanowires grown on top of ITO was investigated using transmission electron microscopy (TEM) characterization. A cross-section TEM sample was prepared using an FEI Helios Nanolab 400s Dual Beam Focused Ion Beam (FIB) SEM. SEM imaging was done using FEI Nova Nano and Zeiss Merlin SEM system. High-resolution transmission electron microscopy (HRTEM) and high-resolution high-angle annular dark-field STEM (HAADF-STEM) characterizations were carried out using a Titan 80-300 ST transmission electron microscope (FEI Company). The elemental composition map was obtained via electron energy loss spectroscopy (EELS).

In order to investigate the polarity of the nanowires, we utilized KOH-based etching. It has been reported that wet chemical etching using KOH shows preferential etching for N-face GaN. Therefore, the polarity can be determined by comparing the morphology of the nanowires before and after KOH etching. We immersed the GaN nanowire on ITO samples within a 40% KOH solution for 1 h in room temperature and compare the morphology before and after chemical immersion to determine the nanowire growth polarity.

We investigated the optical properties of the GaN nanowires directly grown on top of ITO by using a temperature-dependent and power-dependent photoluminescence (PL) measurement setup. The sample was loaded into a helium-cooled cryostat and excited using a 266-nm laser (Teem photonics SNU-20F-10x). The temperature was varied from 10 to 290 K. We first studied the power-dependent photoluminescence response, performed at 10 K. Transmittance measurement was performed using a UV-Vis-NIR spectrophotometer (Shimadzu UV-3600).

X-ray diffraction (XRD) measurement was performed using a Bruker D2 Phaser powder XRD system.

## Results and Discussion

**Fig. 1:**
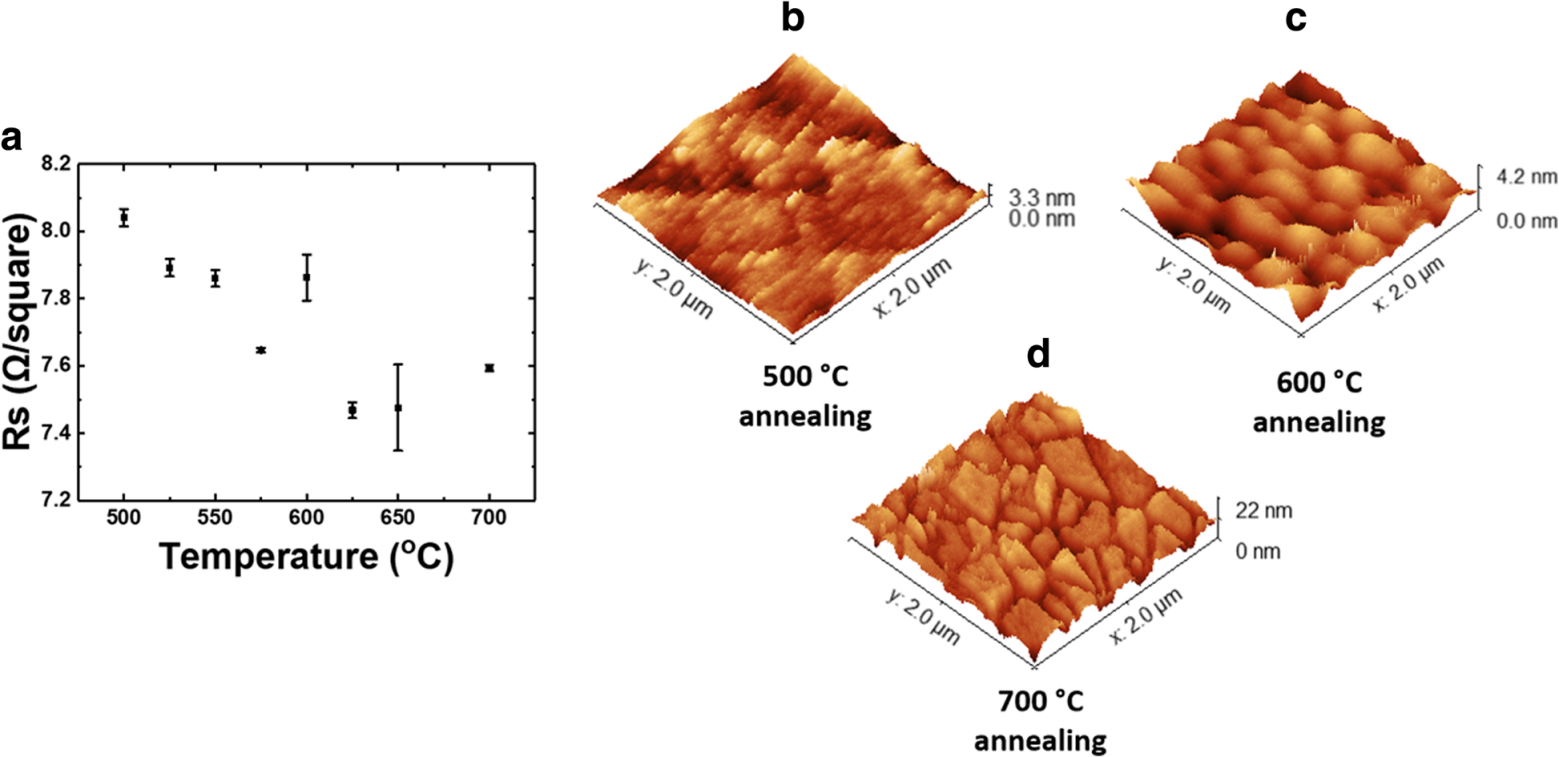
Effect of annealing temperature on the electrical and physical characteristics of deposited ITO thin film. **a** Sheet resistance measured with a four-point probe after annealing at different temperatures. AFM surface topography of the ITO thin film acquired after annealing the sample at **b** 500 °C, **c** 600 °C, and **d** 700 °C

As the high-temperature growth of GaN nanowires might result in the degradation of the underlying ITO layer, we first investigated the effect of thermal annealing on bare ITO deposited on top of the silica substrate. The experiment was performed inside the buffer chamber of the MBE under typically 10^−8^ Torr pressure to simulate actual growth condition. After annealing, the electrical conductivity of the bare ITO is measured using a four-point probe measurement, and the surface roughness is investigated using atomic force microscopy (AFM). From the annealing experiment, shown in Fig. 1a, we find that the value of sheet resistance of the ITO thin film remains below 10 $\Omega / \square $. However, at a higher annealing temperature, the ITO thin film becomes rougher with larger grain size, shown in Fig. 1b–d.

**Fig. 2:**
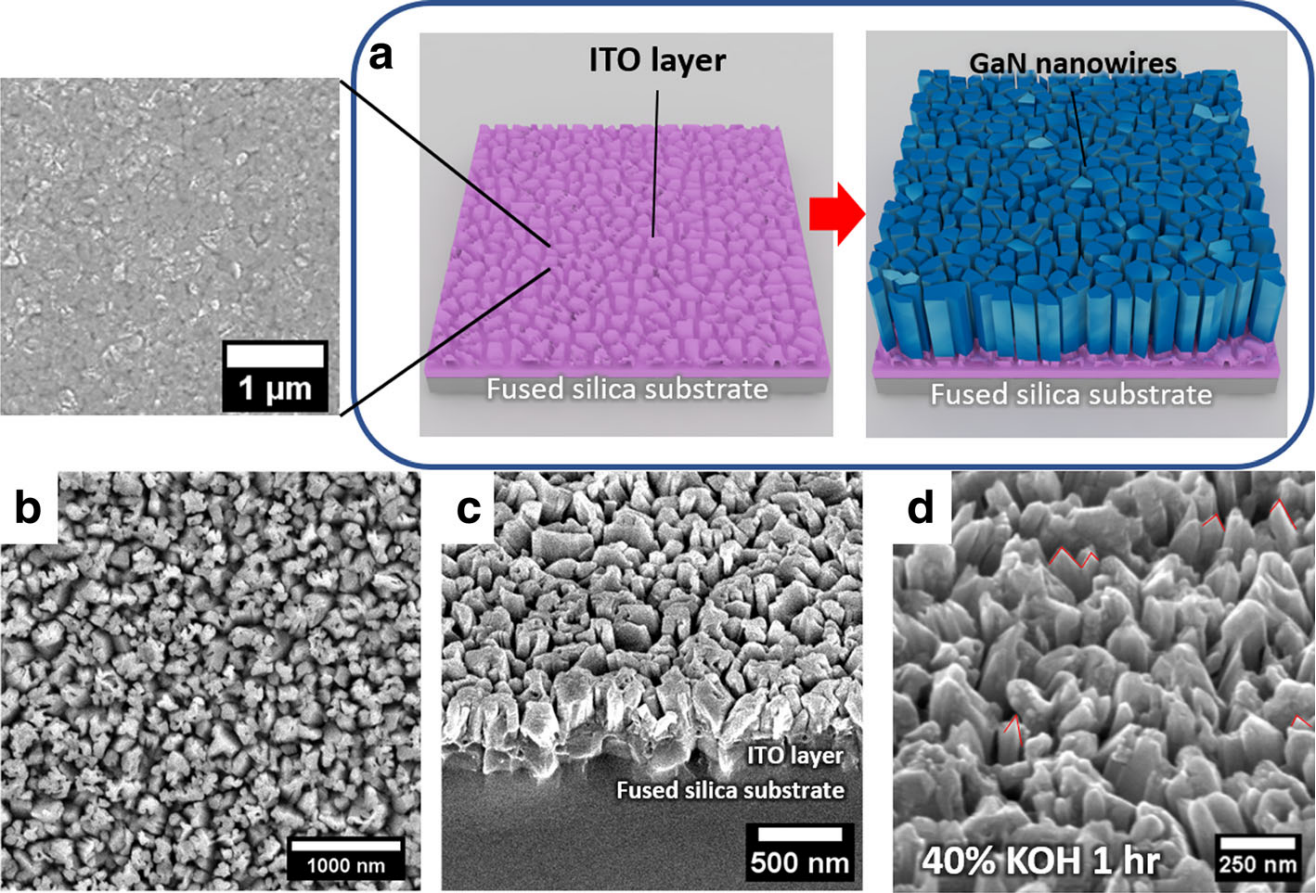
**a** Schematic illustrating the growth of GaN nanowires on rough ITO surface. The inset shows an SEM plan view of the rough ITO surface after thermal annealing. **b** Plan view of GaN nanowires grown on ITO. **c** Elevation view of GaN nanowires grown on ITO. **d** Elevation view of GaN nanowires after 1 h of KOH etching, exposing etched GaN nanowire tip

The nanowire growth process is illustrated in Fig. 2

a. As shown in the AFM result, annealing the ITO layer in elevated temperature will result in rough ITO surface with large grain size. During MBE growth, neighboring GaN nanowires that grow on the surface of a single grain tends to coalesce and form a larger nanowire composed of a cluster of nanowires. Therefore, the morphology of the underlying ITO will directly affect the morphology of the nanowires grown on top of it. The plan view of the scanning electron microscope (SEM) micrograph is shown in Fig. 2b. From the plan view, the nanowire density is statistically estimated to be 9.3×10^9^ cm ^−2^ with a fill factor of 73%. The cross-section view of the sample is shown in Fig. 2c. The nanowires grow perpendicular to the substrate plane with some degree of tilt directly on top of the ITO layer.

The SEM image of the nanowire sample after 1 h of immersion inside 40% KOH solution is shown in Fig. 2d. It can be seen that after the chemical treatment, the tips of the nanowires are partially etched off, which indicates N-polarity. This finding agrees with previously reported results where spontaneously grown III-nitride nanowires are typically N-polar [16–19].

**Fig. 3:**
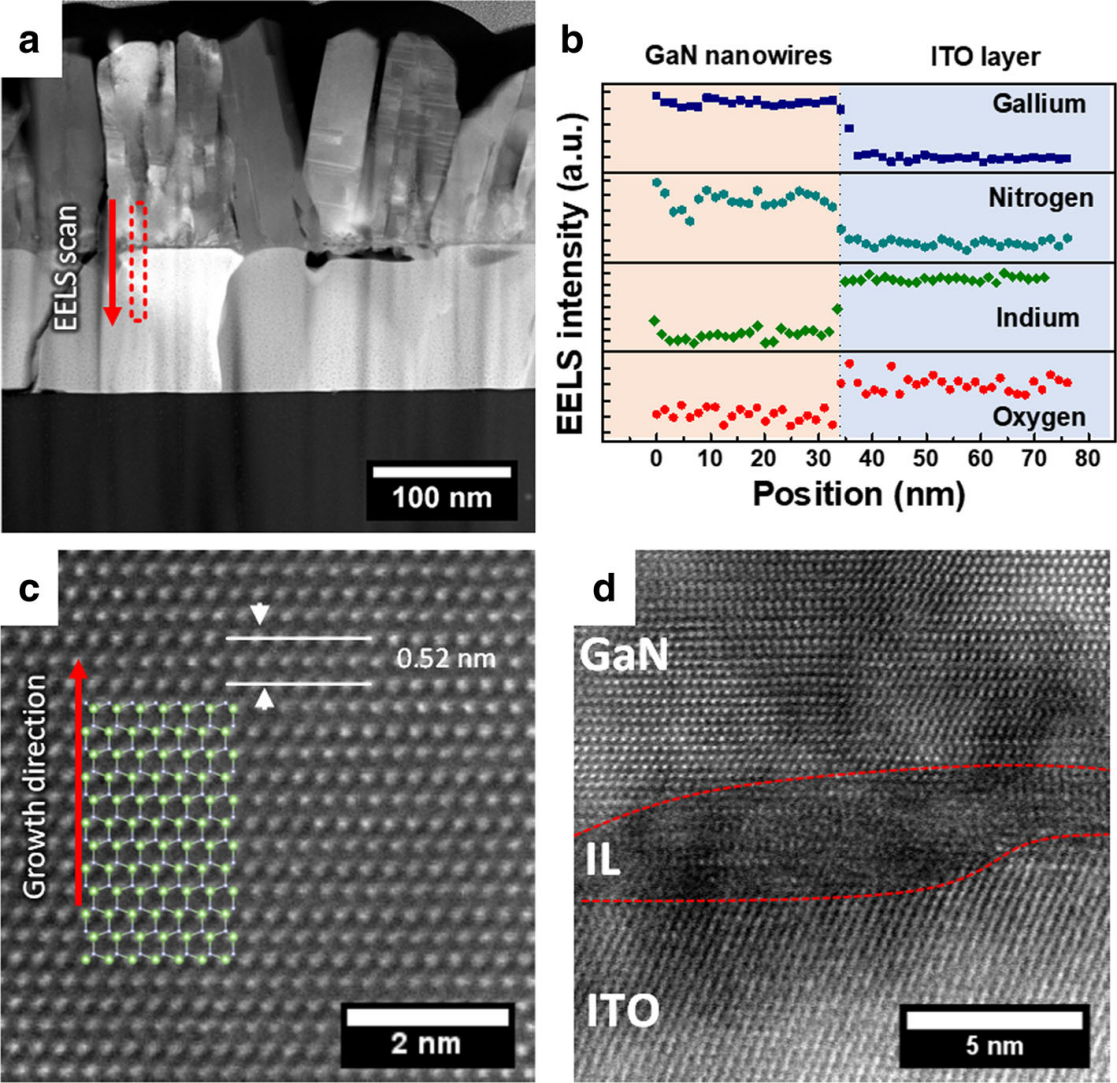
TEM and elemental mapping of GaN nanowires grown on ITO layer. **a** HAADF image of GaN nanowires directly grown on top of the ITO layer. The red box indicates where the EELS line scan was performed. **b** EELS line scan profile of the interface between the base of GaN nanowire and ITO layer. The elemental mapping for Ga, In, N, and O are shown in the graph. **c** High-resolution TEM of the GaN nanowire, showing single crystallinity. The red arrow indicates growth direction. The interplane spacing corresponds to GaN c-plane. **d** High-resolution TEM image of the interface between the GaN nanowire and ITO layer. A partially amorphous intermediate layer (IL) can be seen between the GaN nanowires and ITO layer, bound by the dashed red lines

Figure 3a shows the high-angle annular dark-field scanning transmission electron microscopy (HAADF-STEM) of the nanowires. The nanowires grow directly on top of the ITO layer. To study the elemental composition of the interface between the nanowires and ITO layer, we performed an elemental mapping line scan for Ga, In, N, and O utilizing EELS in the area bound within a red box. The line scan profile is shown in Fig. 3b. The line profile indicates a clear boundary between the GaN nanowires and ITO. A high-resolution TEM image of a single nanowire in Fig. 3c shows the lattice arrangement of the nanowire, confirming the single crystallinity of the material. High-resolution TEM on the interface between GaN nanowires and ITO layer in Fig. 3d shows what appears to be an intermediate layer (IL) composed of a mixture between polycrystalline and amorphous layer about 4 nm thick between the base of the nanowire and the ITO. This thin layer is suggested to be a transition GaN layer, formed between the polycrystalline ITO layer and crystalline GaN layer. A similar layer has been reported before where GaN nanowires are grown directly on top of an amorphous fused silica layer [15].

**Fig. 4:**
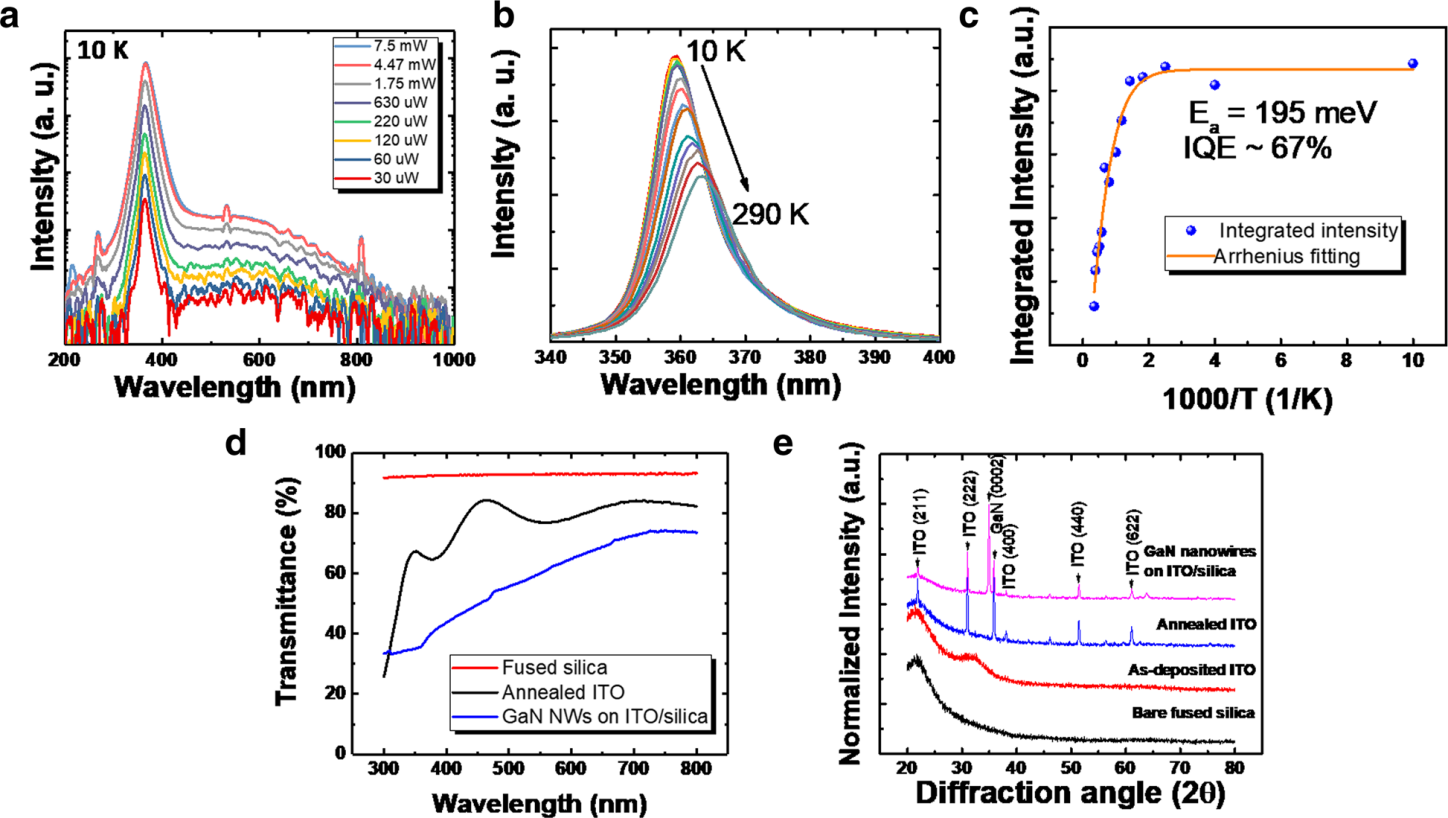
**a** Power-dependent measurement of GaN nanowires grown on Indium Tin Oxide performed at 10 K. **b** Temperature-depende nt PL of GaN nanowires grown on ITO layer. **c** Calculated activation energy based on temperature-dependent PL measurement. **d** Transparency of fused silica substrate, annealed ITO layer on fused silica, and GaN nanowires grown on ITO layer; **e** The XRD profiles for bare fused silica, as-deposited ITO thin film, annealed ITO thin film, and GaN nanowires grown on ITO

The temperature-dependent photoluminescence result is shown in Fig. 4a. From the measurement, it is shown that the yellow luminescence commonly associated with defects in GaN materials is about three magnitudes lower than the GaN band-edge emission, highlighting the high-quality GaN material growth. Temperature-dependent photoluminescence is shown in Fig. 4b. The result shows red-shifting with increasing temperature commonly associated with Varshni band-gap shrinking. The intensity of the peak emission is reduced with the increase in temperature owed to the activation of the non radiative recombination centers. Arrhenius fitting is done on the change in PL integrated intensity over temperature, shown in Fig. 4c. The fitting gives an activation energy of 195 meV. By using the ratio of integrated intensity at 290 K and 10 K, we estimate the internal quantum efficiency of the nanowires to be around 67%.

Figure 4d shows the change in transmittance for the annealed ITO, fused silica, and GaN nanowire on ITO/silica. The transmittance of the sample is reduced after the growth of GaN nanowire. As the GaN nanowires are non-absorbing in the visible wavelength range, the reduced transmittance can be attributed to light scattering caused by the nanowires themselves.

Figure 4e shows the XRD results of the bare silica substrate, silica substrate with as-deposited ITO, RTP-annealed ITO/silica, and GaN nanowires grown on ITO/silica. No XRD peak can be observed in the as-deposited ITO layer, indicating an almost amorphous layer. After RTP annealing, ITO(211), ITO(222), ITO(400), ITO(440), and ITO (622) peaks can be observed, indicating that annealing improves the crystallinity of the ITO layer, which agrees with previous reports [20]. The most dominant peaks are shown to be ITO(222) peak and ITO(400) peak. The measured GaN(0002) peak in the 2 *θ* scan indicates that this plane is parallel to the ITO planes, which shows that the GaN nanowires grow on polycrystalline ITO layer.

**Fig. 5:**
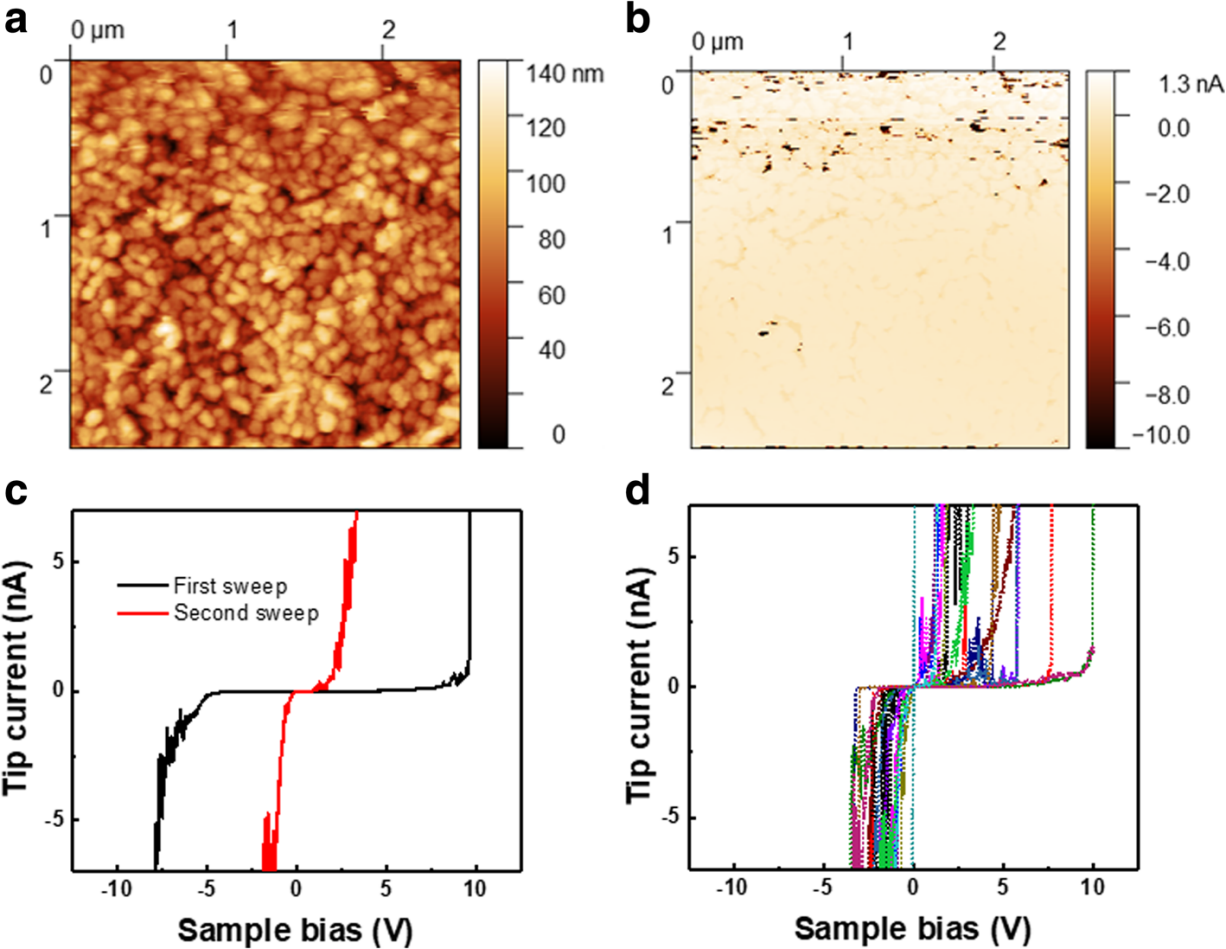
**a** C-AFM mapping of the nanowires topology. **b** Corresponding tip current, with -8V bias applied to the sample. **c** I-V curve of a single nanowire with sample voltage bias from -10 V to 10 V, showing different I-V characteristic between initial and second sweep. **d** Distribution of I-V curve from a number of nanowires, after the initial punch-through sweep

To test whether the GaN nanowires on ITO platform would be feasible for device application, we have grown GaN nanowires with n-doped GaN nanowires using silicon as the dopant and measure the I-V characteristic of individual nanowires using C-AFM. Through this method, we obtained the statistical I-V data from the sample. The resulting measurement is shown in Fig. 5.

The current mapping in Fig. 5b shows that the nanowires in Fig. 5a are initially non-conducting, with only several spots showing current flow. To better investigate why the nanowires are non-conducting, we performed an I-V characterization on individual nanowires. The range of the sample voltage sweep is from − 10 to 10 V, with the resulting tip current ranging from − 10 to 10 nA, which is limited by the AFM system specification. The result is shown in Fig. 5c. For the first sweep, we find that the nanowires exhibit very high turn-on voltage, indicating a Schottky contact behavior between the n-GaN and ITO layer. However, after repeating the measurement, we find that the turn-on voltage of the I-V curve has been significantly reduced, attributed to the lowering of the Schottky barrier height. We observed this trend of reduced turn-on voltage after the initial punch-through voltage sweep across multiple nanowires in the AFM scan area shown in Fig. 5d, confirming that this applies to all of the nanowires grown on ITO. The exact mechanism of the lowering of the turn-on voltage still requires further investigation. Previous reports have shown that applying a high voltage to the material might have induced current-carrying paths through electrical breakdown [21, 22], or modify the structure of the GaN nanowire itself [23] leading to improvement in turn-on voltage.

## Conclusions

In conclusion, we have performed the growth of GaN nanowires on top of an ITO thin film deposited on a fused silica substrate. Physical characterization using electron microscopy shows that the nanowires grow perpendicular to the substrate plane, while retaining high crystal quality. A strong GaN band-edge emission was detected through photoluminescence characterization, while the yellow luminescence commonly associated with defects is absent. The nanowires have a preferred N-polarity, indicated by the preferential etching of the crystal plane in a KOH solution. C-AFM measurements on n-doped nanowires show good conductivity, highlighting the possibility of the platform for device application.

## Abbreviations

AFM: Atomic force microscopy
BEP: Beam equivalent pressure
C-AFM: Conductive atomic force microscopy
EELS: Electron energy loss spectroscopy
FIB: Focused ion beam
IQE: Internal quantum efficiency
HAADF: High-angle annular dark field
HRTEM: High-resolution transmission electron microscopy
ITO: Indium tin oxide
MOCVD: Metal organic chemical vapor deposition
PA-MBE: Plasma-assisted molecular beam epitaxy
PECVD: Plasma-enhanced chemical vapor deposition
PL: Photoluminescence
RF: Radio frequency
RTA: Rapid thermal annealing
sccm: Standard cubic centimeter per minute
SEM: Scanning electron microscopy
STEM: Scanning transmission electron microscopy
TEM: Transmission electron microscopy
XRD: X-ray diffraction
